# Guideline adherence among prehospital emergency nurses when caring for patients with chest pain: a prospective cohort study

**DOI:** 10.1186/s13049-021-00972-5

**Published:** 2021-10-30

**Authors:** Kristoffer Wibring, Markus Lingman, Johan Herlitz, Lina Blom, Otto Serholt Gripestam, Angela Bång

**Affiliations:** 1 Department of Ambulance and Prehospital Care, Region Halland, Varlabergsvägen 29, 434 39 Kungsbacka, Sweden; 2 Institute of Health and Care Sciences, Sahlgrenska Academy, University of Gothenburg, Göteborg, Sweden; 3 Department of Molecular and Clinical Medicine/Cardiology, Institute of Medicine, Sahlgrenska Academy, University of Gothenburg, Göteborg, Sweden; 4 Department of Development, Halland Hospital, Halmstad, Sweden; 5 The Prehospital Research Center Western Sweden, University of Borås, Borås, Sweden; 6 Department of Emergency Care, Södra Älvsborgs Hospital, Region Västra Götaland, Sweden

**Keywords:** Chest pain, Emergency medical services, Acute myocardial infarction, Guideline adherence, Guideline compliance

## Abstract

**Background:**

The emergency medical services (EMS) use guidelines to describe optimal patient care for a wide range of clinical conditions and symptoms. The intent is to guide personnel to provide patient care in line with best practice. The aim of this study is to describe adherence to such guidelines among prehospital emergency nurses (PENs) when caring for patients with chest pain.

**Objective:**

To describe guideline adherence among PENs when caring for patients with chest pain. To investigate whether guideline adherence is associated with patient age, sex or final diagnosis of acute myocardial infarction on hospital discharge.

**Methods:**

Guideline adherence in terms of patient examination and pharmaceutical treatment was analysed in a cohort of 2092 EMS missions carried out in 2018 in Region Halland, Sweden. Multivariate regression was used to describe how guideline adherence is associated with patient age, sex and diagnosis on hospital discharge.

**Results:**

Guideline adherence was high regarding examination of vital signs (93%) and electrocardiogram (ECG) registration (96%) but lower in terms of pharmaceutical treatment (ranging from 28 to 90%). Adherence was increased in cases in which the patient ended up with acute myocardial infarction (AMI) as diagnosis on discharge. Patients with AMI were given acetylsalicylic acid by PENs in 50% of cases. Women were less likely than men to receive treatment with acetylsalicylic acid and oxycodone.

**Conclusions:**

Guideline adherence among PENs when caring for patients with chest pain is satisfactory in terms vital signs and ECG registration. Regarding pharmaceutical treatment guideline adherence is defective. Improved adherence is mainly associated with male sex in patients and a diagnosis of AMI on hospital discharge. Defective adherence excludes measures known to improve patients’ prognoses such as treatment with acetylsalicylic acid.

## Background

Chest pain is one of the most common complaints when contacting the emergency medical services (EMS). About 10–15% of all patient-related EMS missions concern patients with chest pain [1, 2] out of which about 10% have an acute myocardial infarction (AMI) [3, 4].

The prehospital care of patients with acute non-traumatic chest pain is well established. It includes examination of vital signs and ECG registration, treatment with acetylsalicylic acid, glyceryl trinitrate and morphine [5]. If ST-elevation is present on the ECG, appropriate reperfusion should be initiated with minimal delay [6].

The EMS use guidelines to describe optimal patient care for a wide range of clinical conditions and symptoms, including chest pain. The intent is to guide EMS personnel to provide patient care in line with best practice [7]. However, guideline adherence among prehospital personnel varies widely between different studies and organisations [7–10]. In general, guideline adherence is reported to be fairly low [7–10]. Unsatisfactory guideline adherence deprives patients of best practice care and may result in avoidable morbidity or even death.

The poor guideline compliance in previous studies is partly explained by medication contraindications such as allergies or patients’ previous medical history. However, this only explains a small proportion of cases of noncompliance [8]. Instead, low adherence is mainly explained either by the EMS personnel’s assessment of chest pain as non-cardiac [9, 11] or of a patient’s condition as being low priority [9].

This study describes chest pain guideline adherence among prehospital emergency nurses (PENs) and whether adherence varies depending on patient sex, age or prevalence of AMI.

## Objective

- To describe guideline adherence among prehospital emergency nurses when caring for patients with chest pain.
- To investigate whether guideline adherence is associated with patient age, sex or final diagnosis of AMI on hospital discharge.

## Methods

The study is part of the BRIAN research programme ((BRöstsmärta I AmbulaNs (Swedish), EMS Chest pain (English)). The BRIAN programme is mainly focused on prehospital assessment of patients with chest pain. Study population, data collection and clinical setting have been previously described and are therefore summarised briefly [4].

### Hypothesis

The hypothesis was that adherence to guidelines would be associated with the final diagnosis (to some extent reflecting the estimated risk already on scene). Furthermore, we hypothesised that sex and age may be influential factors since both may influence the degree of suspicion of AMI (increased suspicion among men and among the elderly).

### Study population

**Fig. 1:**
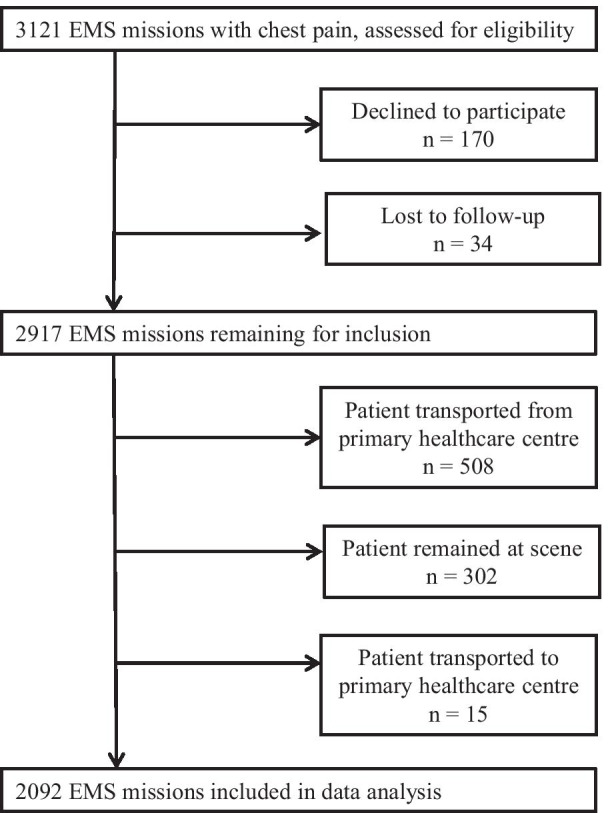
The number of EMS missions excluded and for what reason

A total of, 3121 EMS missions were carried out in the county catchment area including patients ≥ 18 years old and with a chief complaint of chest pain (assigned Rapid Emergency Triage and Treatment System (RETTS) code 5, i.e. chest pain, according to PEN on scene [12]). All these missions were eligible for inclusion. After excluding patients declining to participate and patients who were lost to follow-up, 2917 EMS missions remained. Of these, 508 concerned transport from a primary healthcare centre to hospital (Fig. 1). These EMS missions were excluded since in these cases the general practitioner is responsible for patient care during transport and the EMS guidelines do not apply.

Furthermore, 302 and 15 EMS missions respectively were excluded since the patients remained on scene or were transported to a primary healthcare centre (Fig. 1). These cases represent patients for whom the need of care was assessed by the PEN as low or non-existent and also cases in which the patients opposed care.

### Clinical setting

The study was conducted in the county of Halland, Sweden in 2018. Halland has an integrated healthcare system with a catchment area of 5500 km^2^ and with 329,000 inhabitants in 2018. These are served by two emergency hospitals, including one with percutaneous coronary intervention (PCI) capabilities. There is a single, tax funded, one-tiered EMS organisation with eight ambulance stations and 19 ambulances active during daytime. In 2018, a total of 30,000 missions were carried out by the EMS (inter-hospital site transports excluded).

### EMS chest pain guidelines

In 2018, the EMS guidelines of the county studied stated that all patients with non-traumatic chest pain should be:

- examined using:
  - complete vital signs (oxygen saturation, breathing rate, heart rate, systolic blood pressure, level of consciousness and body temperature)
  - ECG
- treated with:
  - acetylsalicylic acid if pain of suspected cardiac origin
  - oxycodone if pain rated > 3 according to Numeric Rating Scale (NRS)
  - sublingual glyceryl trinitrate if pain rated > 3 according to NRS
  - ondansetron if nausea
  - oxygen if oxygen saturation < 90%

### Data collection

Each patient was traced throughout the healthcare chain, from EMS mission to hospital discharge. Data on demographics, vital signs, ECG and medical treatment was retrieved from the EMS medical record. Data on symptoms was collected using a novel questionnaire [4] embedded in the EMS medical record. By using electronic tablets, both the questionnaire and the EMS record were available at the bedside during the entire EMS mission. Diagnosis on hospital discharge, according to physician in charge, was retrieved from the hospital medical record.

### Endpoint

Guideline adherence was defined as:

1. complete vital signs
2. ECG registration
  Treated with:
3. acetylsalicylic acid
4. oxycodone
5. sublingual glyceryl trinitrate
6. ondansetron
7. oxygen

### Statistical analysis

The results are presented using descriptive statistics including percentage (%), number of patients (n), median and quartiles where appropriate.

Differences in guideline adherence with respect to patient age, sex and a diagnosis of AMI on hospital discharge were analysed using univariate logistic regression. When analysing the association between age and guideline adherence, the cohort was divided into two groups using median age as cut-off.

Guideline adherence association with patient age, sex or diagnosis of AMI on hospital discharge was thereafter analysed with multivariate logistic regression to provide adjusted results. One multivariate analysis was executed for each of the seven endpoints described above. *P*-values below 0.05 were considered statistically significant. All analyses were carried out using IBM SPSS Statistics 27.

Missing data on ECG, vital signs and pharmaceutical treatment was equated to not being provided as the personnel only registered data on executed tasks. If data was missing on age, sex or diagnosis on hospital discharge the EMS mission was not included in the study. EMS missions with missing data on pain intensity and nausea were excluded from the univariate analyses on treatment with oxycodone and sublingual glyceryl trinitrate respectively ondansetron. For the multivariate analyses data on symptoms were not included and was executed on the complete cohort.

## Results

In total, 2092 EMS missions concerning 1680 unique patients were included. The median age of the cohort was 73 years old (Q25–Q75, 60–83). Sex was evenly distributed. The prevalence of AMI at hospital discharge was 12%. The corresponding figure for male patients were 15% and for female patients 9%. A majority of the patients rated their pain as three or higher according to NRS. Almost one third of the patients stated they were nauseous. Oxygen saturation below 90% was present in only three percent of the EMS missions (Table 1).

**Table 1:** Description of study sample

	All	Sex		Acute myocardial infarction		Missing
		Women	Men	Yes (%)	No (%)	
All (%)	2092 (100)	1054 (50)	1038 (50)	254 (12)	1838 (88)	0 (0)
*Age*						
Median (Q25–Q75)	73 (60–83)	74 (62–84)	72 (58–82)	76 (67–85)	73 (58–83)	0 (0)
Acute myocardial infarction (%)	254 (12)	94 (9)	160 (15)	254 (100)	0 (0)	0 (0)
NRS > 3 n (%)	1234 (65)	620 (65)	614 (65)	160 (72)	1074 (64)	196 (9)
Nausea (%)	540 (30)	305 (33)	235 (26)	63 (31)	477 (29)	270 (13)
Oxygen saturation < 90% (%)	60 (3)	28 (3)	32 (3)	13 (5)	47 (3)	4 (0.2)
*Priority by emergency dispatch centre*						0 (0)
Priority 1	1452 (69)	717 (68)	735 (71)	199 (78)	1253 (68)	
Priority 2	628 (30)	328 (31)	300 (29)	53 (21)	575 (31)	
Priority 3	12 (1)	9 (1)	(3 (< 1)	2 (1)	10 (1)	
Priority 4	0 (0)	0 (0)	0 (0)	0 (0)	0 (0)	
*Priority by EMS-personnel*						
Priority 1	242 (12)	81 (8)	161 (16)	98 (39)	144 (8)	0 (0)
Priority 2	1606 (77)	824 (78)	782 (75)	142 (56)	1464 (80)	
Priority 3	238 (11)	145 (14)	93 (9)	14 (6)	224 (12)	
Priority 4	6 (< 1)	4 (< 1)	2 (< 1)	0 (0)	6 (< 1)	
Median EMS time with patient (min) (Q1–Q3)	50 (44–65)	52 (34–68)	50 (35–63)	52 (48–68)	50 (33–65)	27 (1)

Description of the complete cohort of 2092 patients

NRS, Numeric Rating Scale, EMS, Emergency Medical Services

Guideline adherence was above 90% regarding ECG registration and complete examination of vital signs, while adherence to pharmaceutical treatment was considerably lower with the exception of oxygen administration. Acetylsalicylic acid was provided in 28% of all patients and to 50% of patients with AMI. For patients with pain > 3 according to NRS 53 percent were treated with sublingual glyceryl trinitrate and 39% with oxycodone (Table 2).

**Table 2:** Associations between guideline adherence and patient age, sex and diagnosis of AMI (univariate analyses)

	All (%)	Age (%)		*P*-value	Odds ratio confidence interval 95%	Sex (%)		*P*-value	Odds ratio confidence interval 95%	Acute myocardial infarction (%)		*P*-value	Odds ratio confidence interval 95%
		≤ 73	> 73			Women	Men			Yes	No		
Complete vital signs registered	1950 (93)	970 (92)	980 (94)	0.031	1.04–2.07	988 (94)	962 (93)	0.336	0.60–1.19	231 (91)	1719 (94)	0.127	0.44–1.11
ECG registered	2011 (96)	1008 (96)	1003 (97)	0.241	0.84–2.05	1024 (97)	987 (95)	0.015	0.36–0.90	249 (98)	1762 (96)	0.101	0.86–5.36
*Medical treatment*													
Oxygen^a^	54 (90)	8 (89)	46 (90)	0.904	0.12–11.18	26 (93)	28 (88)	0.495	0.09–3.19	10 (77)	44 (94)	0.095	0.04–1.30
Acetylsalicylic acid	587 (28)	311 (39)	276 (27)	0.138	0.715–1.05	253 (24)	334 (32)	< 0.001	1.24–1.82	127 (50)	460 (25)	< 0.001	2.29–3.92
Sublingual glyceryl trinitrate^b^	659 (53)	354 (53)	305 (54)	0.708	0.83–1.31	323 (52)	336 (55)	0.355	0.89–1.39	95 (59)	564 (53)	0.105	0.94–1.85
Oxycodone^b^	484 (39)	255 (38)	229 (41)	0.387	0.88–1.39	212 (34)	272 (44)	< 0.001	1.22–1.93	87 (54)	397 (37)	< 0.001	1.45–2.84
Ondansetron^c^	177 (33)	101 (31)	76 (36)	0.246	0.86–1.79	95 (31)	82 (35)	0.358	0.83–1.70	33 (52)	144 (30)	0.001	1.50–4.33

Univariate logistic regression analyses. Analyses on treatment with oxygen, sublingual glyceral trinitrate, oxycodone and odansetron excuted on subgroups based on signs and symptoms as stated below

ECG = Electrocardiogram

^a^ if oxygen satuartion < 90% (60 patients)

^b^ if patient rated pain according to NRS > 3 (1234 patients)

^c^ if patient reported nausea (540 patients)

When adjusting for sex and AMI older patients more commonly had all their vital signs examined compared to younger patients (odds ratio (OR) 1.5). They were also more often given oxygen (OR 2.0) while treatment with ondansetron was rarer (OR 0.72) (Table 3).

**Table 3:** Assocations between guideline adherence and patient age, sex and diagnosis of AMI (multivariate analyses)

	Age > 73			Male sex			Acute myocardial infarction		
	*P*-value	Odds ratio	Confidence interval, 95%	*P*-value	Odds ratio	Confidence interval, 95%	*P*-value	Odds ratio	Confidence interval, 95%
Complete vital signs registered	0.026	1.49	1.05–2.11	0.504	0.89	0.63–1.26	0.105	0.68	0.42–1.09
ECG registered	0.358	1.24	0.79–1.94	0.011	0.55	0.35–0.87	0.076	2.30	0.92–5.76
*Medical treatment*									
Oxygen	< 0.001	2.05	1.62–2.60	0.461	1.09	0.57–1.37	< 0.001	2.22	1.64–2.99
Acetylsalicylic acid	0.052	0.82	0.68–1.00	0.001	1.39	1.14–1.69	< 0.001	2.94	2.24–3.86
Sublingual glyceryl trinitrate	0.429	0.93	0.78–1.11	0.839	0.98	0.82–1.17	< 0.001	1.65	1.27–2.16
Oxycodone	0.411	0.92	0.76–1.12	0.001	1.40	1.14–1.70	< 0.001	2.12	1.61–2.78
Ondansetron	0.016	0.72	0.55–0.94	0.253	0.86	0.66–1.12	< 0.001	2.42	1.73–3.40

Multivariate logistic regression analyses including age, sex and diagnosis of AMI on hospital discharge. Analyses excuted on the complete cohort, one analysis for each of the seven guideline objects

*ECG* Electrocadiogram

Male patients, when adjusting for age and AMI, were less likely to have an ECG registered than female patients (OR 0.55). Both acetylsalicylic acid (OR 1.4) and oxycodone (OR 1.4) were significantly more often administered to men than to women (Table 3).

A diagnosis of AMI on hospital discharge was associated with higher rates of pharmaceutical treatment when adjusting for sex and age. This accounts for all drugs stated in the EMS guidelines and especially when it comes to acetylsalicylic acid (OR 2.9). AMI on hospital discharge did not affect the probability of ECG registration or complete examination of vital signs (Table 3).

**Fig. 2:**
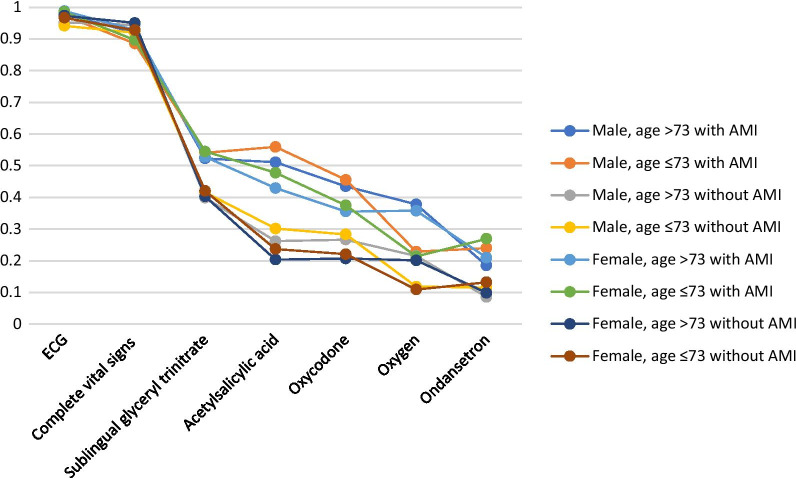
Probability of guideline adherence based on the models provided by the multivariate analyses

The probability of the PEN providing medical treatment was mainly associated with diagnosis of AMI as visualised in Fig. 2. Patient characteristics in terms of age and sex only had little impact on the probability of the PEN measuring vital signs and registering ECG. Sex was associated with the probability of pharmaceutical treatment with women having a lowered probability compared to men (Fig. 2).

## Discussion

This study shows that ECG registration and examination of vital signs were carried out to a large extent by PENs when caring for patients with chest pain. On the other hand, guideline adherence was fairly low in terms of pharmaceutical treatment. This is in line with previous studies investigating guideline adherence among EMS personnel, both PENs and paramedics [7–10]. As hypothesised, improved guideline adherence was associated with a final diagnosis of AMI along with male sex. However, association with patient sex remained also after adjusting for final diagnosis of AMI which was not expected. In the adjusted analyses, old age was associated with improved guideline adherence in terms vital signs and treatment with oxygen, but older patients received ondansetron to a lesser extent.

The lack of guideline adherence in terms of medication administration can probably to some extent be explained by medication contraindications, lack of time when caring for severely ill patients during short transport times or patient refusal. However, previous research states that contraindications are rare [13]. Furthermore, only 12% of EMS missions included were transported with the highest priority to hospital with a median patient care time of 50 min (Table 1). This indicate that rapid patient transport with lights and sirens was rarely needed according to the PEN and that time was not a limiting factor for drug administration in most cases. In addition, patients who refuse medication are also likely to refuse conveyance and these cases were excluded from the study. We may therefore conclude that the situations described are rare and are therefore not the main reasons for omitting to administer the recommended drugs. These situations do not explain either why adherence varies above all with a diagnosis of AMI on hospital discharge, but also in relation to patient sex and age.

Finding that patients with AMI to a larger extent receive medication in line with guidelines indicates that PENs improve their adherence when they suspect the chest pain being of acute cardiac origin. This is also in line with previous results [9, 11]. In one way this is a positive result since it indicates that PENs to some degree manage to distinguish those with AMI as opposed to those with other origins of their chest pain. However, this approach of basing medical treatment on PENs suspicion of AMI results in 50% of the patients with AMI ending up without prehospital medication with acetylsalicylic acid. This may be particularly problematic since early treatment with acetylsalicylic acid when suffering from AMI has been reported to decrease mortality rates [5]. We suggest altering the guidelines to support medication with acetylsalicylic acid also when suspicion of ACS is low to increase the proportion of patients with AMI provided acetylsalicylic acid already by the EMS.

In our study women are less likely than men to receive treatment with acetylsalicylic acid and oxycodone. These differences are at hand both in the univariate analyses and multivariate ones adjusting for age and diagnosis of AMI on hospital discharge. The difference can thereby not be explained by neither the higher median age nor the lower incidence of AMI among women but rather by sex itself. This is problematic as it indicates an inequality in EMS care of patients with chest pain based on sex. Sex inequalities in AMI care has been shown in previous in-hospital research [14–16] and also regarding direct admission to PCI [17] and time to acetylsalicylic acid treatment [18]. But, to the best of our knowledge, it has not previously been reported that women with chest pain are less likely than men to receive pharmaceutical treatment already in the prehospital phase. The occurrence of sex differences in prehospital chest pain care should be investigated further along with research on why such differences exists.

An ECG was registered in 96% of all cases. Thus this is the part of the guidelines with the highest adherence among PENs. Previous research shows an association between underuse of ECG and increased mortality [19]. The high adherence is a positive finding and this should benefit the patient.

A guideline adherence of 100% is neither possible nor desirable given the multifaceted setting in which prehospital emergency care takes place. Patient characteristics, lack of time, limited personnel resources and technical errors are all acceptable reasons why adherence to guidelines is not always possible. However, in most cases, the conditions do not prohibit guideline adherence and improved compliance should be possible. Two easily accessible ways to achieve this may be firstly to advocate a more generous approach regarding both examination and pharmaceutical treatment, and secondly that the guidelines should apply also in cases in which PENs judge the probability of cardiac origin to be low. Improving both EMS and the emergency dispatch centre personnel ability to identify patients with AMI by the use of risk assessment tools [20, 21] and/or introducing prehospital troponin [21–24] could also be ways to enhance the prehospital care of patients with chest pain, not only in terms of guideline adherence. Improved adherence may also possibly be achieved by using interactive tools that suggest appropriate examination and treatment based on the data the EMS personnel feed into the tools [25]. Furthermore, education of EMS personnel on the difficulties of ruling out AMI in the EMS setting, along with structured follow-up of in-hospital patient care, could give rise to new insights and maybe a more generous approach when caring for patients with chest pain. Studies on how to improve guideline adherence among EMS personnel are warranted.

## Strengths and limitations

This study is strengthened by the unselected inclusion of EMS missions comprising an almost complete population of EMS chest pain patients during one year. It investigates guideline adherence both in terms of patient examinations and various medical treatments and how this is associated with diagnosis on discharge which, to our knowledge, has not been studied before in the prehospital context.

One limitation is that the multivariate analyses do not take into account whether the medical treatment provided was called for or not in terms of presence of nausea, desaturation or pain rated higher than three according to NRS. These aspects were only considered in the univariate analyses. This distinction was necessary to achieve statistical viability but complicates comparison between the uni- and multivariate analyses. Such “over treatment” can be considered a breach of the guidelines. However, the drugs included in the current guidelines are not very potent and “over treatment” is therefore quite unproblematic. For example studies has shown that short term “over treatment” with oxygen do not affect patient outcome [26]. Furthermore, prehospital treatment with acetylsalicylic acid [27] or sublingual glyceryl trinitrate [28] are rarely associated with adverse events. In addition, the identified associations between guideline adherence and age, sex and AMI are of interest regardless of if “over treatment” is at hand or not.

This study is partly based on data retrieved from the EMS records. It is possible that PENs sometimes failed to document examinations carried out and medication administered. Thus guideline adherence may actually be better than can be inferred from available data. The prevalence of such documentation error is unknown. Documentation in the EMS record was possible both bedside and during transport by using tablets, as well as after completed mission by computers at the emergency department and the EMS stations. Furthermore, PENs are obliged by Swedish law to document which examinations they carry out and which medication they administer. It is therefore assumed that such documentation errors are rare and do not affect the results to any significant extent. There is also a risk that the PENs register tasks that were not actually performed or improved their guideline adherence since the PENs were aware that data from the EMS record was to be used in the current study. Guideline adherence may, thereby, also be lower than the results indicate. It is difficult to assess if, and if so, to what extent this is at hand, but the risk should not be neglected. Both missed registrations and registering tasks that were not executed affects the internal validity negatively.

This study only considers patient related factors when analysing association with guideline adherence. However, guideline adherence is probably associated with several other, non-patient, factors such as education and experience of the PEN, time of the day, workload and distance to hospital. It was not within the scope of this study to investigate such factors, but one should be aware that other variables than the ones studied may influence guideline adherence. This negatively affect the external validity and should be considered when applying the results on other EMS organisations and settings with other prerequisites regarding for example EMS personnel education or EMS funding. The findings of this study primarily apply to EMS organisations with PENs staffing the ambulances. As the findings, in terms of adherence to pharmaceutical treatment guidelines, confirm the findings from previous studies [7–10] on both nurse and paramedic guideline adherence it is reasonable to assume that EMS personnel education level is not crucial for guideline adherence. Furthermore, the nearly complete and unselected inclusion of all EMS missions within the county concerning patients with chest pain during 2018 and data being provided by close to 200 different PENs increases the generalisability of the results.

In this study the same patient could be included several times if cared for by the EMS on multiple occasions during the inclusion period (2092 EMS missions concerning 1680 unique patients). Therefore, one can argue that included EMS missions are not independent events. However, caring for the same patient on multiple occasions reflect the reality of EMS care and guidelines do not state that patients should be cared for differently based on the quantity of EMS missions. Furthermore, there are close to 200 PENs working in the current county. This lowers the probability for same PEN caring for the same patient on multiple occasions. We therefore judged it appropriate not to exclude patients based on number of EMS contacts.

## Conclusions

Guideline adherence among PENs when caring for patients with chest pain is satisfactory in regarding registration of vital signs and ECG. Guideline adherence among PENs, in terms of medical treatment, is defective. Adherence is better in cases in which patients are diagnosed with AMI on hospital discharge. This indicates that PENs’ assessments of patients’ conditions affect which medications patients receive, which is positive. However, this results in only 50% of the patients diagnosed with AMI on hospital discharge receiving acetylsalicylic acid in the prehospital setting. Improved adherence is associated with higher age and male sex indicating age, but above all, gender inequalities in EMS care of patients with chest pain.

## Data Availability

The datasets generated and analysed during the current study are not publicly available due the integrity of patient privacy but are available from the corresponding author on reasonable request and if approved by the Regional Ethical Review Board in Lund.

## Abbreviations

AMI: Acute myocardial infarction
ECG: Electrocardiogram
EMS: Emergency medical services
NRS: Numeric rating scale
OR: Odds ratio
PCI: Percutaneous coronary intervention
PEN: Prehospital emergency nurse
